# Prediction of hospital-acquired influenza using machine learning algorithms: a comparative study

**DOI:** 10.1186/s12879-024-09358-1

**Published:** 2024-05-02

**Authors:** Younghee Cho, Hyang Kyu Lee, Joungyoun Kim, Ki-Bong Yoo, Jongrim Choi, Yongseok Lee, Mona Choi

**Affiliations:** 1 College of Nursing, Yonsei University, Seoul, Republic of Korea; 2 Department of Digital Health, Samsung SDS, Seoul, Republic of Korea; 3 College of Engineering, University of Seoul, Seoul, Republic of Korea; 4 Division of Health Administration, Yonsei University, Wonju, Republic of Korea; 5 College of Nursing, Keimyung University, Daegu, Republic of Korea; 6 Mo-Im Kim Nursing Research Institute, College of Nursing, Yonsei University, 50 Yonsei-ro, Seodaemun-gu, Seoul, 03722 Republic of Korea

**Keywords:** Influenza, Human, Cross infection, Machine learning, Logistic models, Random forest, Patient’s rooms

## Abstract

**Background:**

Hospital-acquired influenza (HAI) is under-recognized despite its high morbidity and poor health outcomes. The early detection of HAI is crucial for curbing its transmission in hospital settings.

**Aim:**

This study aimed to investigate factors related to HAI, develop predictive models, and subsequently compare them to identify the best performing machine learning algorithm for predicting the occurrence of HAI.

**Methods:**

This retrospective observational study was conducted in 2022 and included 111 HAI and 73,748 non-HAI patients from the 2011–2012 and 2019–2020 influenza seasons. General characteristics, comorbidities, vital signs, laboratory and chest X-ray results, and room information within the electronic medical record were analysed. Logistic Regression (LR), Random Forest (RF), Extreme Gradient Boosting (XGB), and Artificial Neural Network (ANN) techniques were used to construct the predictive models. Employing randomized allocation, 80% of the dataset constituted the training set, and the remaining 20% comprised the test set. The performance of the developed models was assessed using metrics such as the area under the receiver operating characteristic curve (AUC), the count of false negatives (FN), and the determination of feature importance.

**Results:**

Patients with HAI demonstrated notable differences in general characteristics, comorbidities, vital signs, laboratory findings, chest X-ray result, and room status compared to non-HAI patients. Among the developed models, the RF model demonstrated the best performance taking into account both the AUC (83.3%) and the occurrence of FN (four). The most influential factors for prediction were staying in double rooms, followed by vital signs and laboratory results.

**Conclusion:**

This study revealed the characteristics of patients with HAI and emphasized the role of ventilation in reducing influenza incidence. These findings can aid hospitals in devising infection prevention strategies, and the application of machine learning-based predictive models especially RF can enable early intervention to mitigate the spread of influenza in healthcare settings.

**Supplementary Information:**

The online version contains supplementary material available at 10.1186/s12879-024-09358-1.

## Background

Hospital-acquired influenza (HAI) is associated with significant morbidity and mortality, leading to extended hospital stays and increased medical costs. Studies have shown that a quarter of all influenza cases among hospitalized patients can be attributed to HAI [1]. Mortality rates range from 9% [1] to 18.8% [2], with a high prevalence of 39.2% in critically ill patients [3]. Nevertheless, most healthcare providers consider influenza a community-acquired infection, and HAI is under-recognized because patients are discharged before being diagnosed with influenza due to the incubation period [4]. However, HAI patients have longer hospital and intensive care unit lengths of stay (LoS) [2–6] and higher mortality rates than community-acquired influenza (CAI) patients [2, 3, 5, 7, 8]. In addition, the poor outcomes of HAI require medical resources that could be used to treat other patients.

Inpatients can acquire influenza through direct or indirect contact with infected family members, visitors, healthcare personnel, and fellow patients [9]. Multi-occupancy rooms with an average of 4.2 beds per room are common in South Korea, constituting 77% of rooms in tertiary hospitals and 79% in general hospitals [10]. It is customary for family members or professional caregivers to stay with patients in hospital rooms for care, and frequent visits are widespread. As a result, patients face an increased susceptibility to influenza infection in such environments. Additionally, influenza has an incubation period and is most contagious for 3–4 days after symptom onset. Some individuals transmit the virus with minimal or no symptoms, leading to influenza outbreaks in hospital settings [11]. Therefore, it is crucial for clinicians to promptly identify influenza infections, regardless of whether patients exhibit symptoms, and to administer preventive care to infected patients.

Conversely, electronic medical record (EMR) integration into hospitals allows the real-time collection of a diverse range of patient data, facilitating machine learning (ML) algorithm applications in medical contexts for proactive prognosis and disease onset prediction [12–21]. ML, a subset of artificial intelligence (AI), analyses historical datasets, creating predictive models from raw data to advance evidence-based medicine, including risk analysis, screening, prediction, and personalized care [20, 22]. ML algorithms reduce uncertainty and enhance clinical decision-making to improve patient outcomes and quality [17, 18]. Previous studies have successfully constructed prediction models for various conditions, such as acute graft-versus-host disease (GVDH) [12], recurrent clostridium difficile infection (rCDI) [21], sepsis [15, 16], and mortality risk [17, 19]. To our knowledge, no studies have been conducted on developing predictive models for HAI.

This study aimed to investigate the key factors associated with HAI. Subsequently, the essential features were identified and utilized as inputs for four distinct ML algorithms in developing predictive models. Finally, the performance of the models was assessed and compared, leading to the identification of the most effective ML algorithm for accurately predicting HAI occurrence.

## Methods

### Study design and setting

This was a retrospective, observational, single-centre study using EMR data. The dataset was obtained from the Yonsei University Health System, a tertiary teaching hospital in Seoul, South Korea. The study was conducted in 2022 and encompassed the influenza seasons spanning from 2011 to 2012 to 2019–2020, covering the months from October to April of the subsequent year. The exclusion of March and April 2020 from the 2019–2020 season was justified by the onset of the COVID-19 pandemic in March 2020.

### Study population

**Fig. 1:**
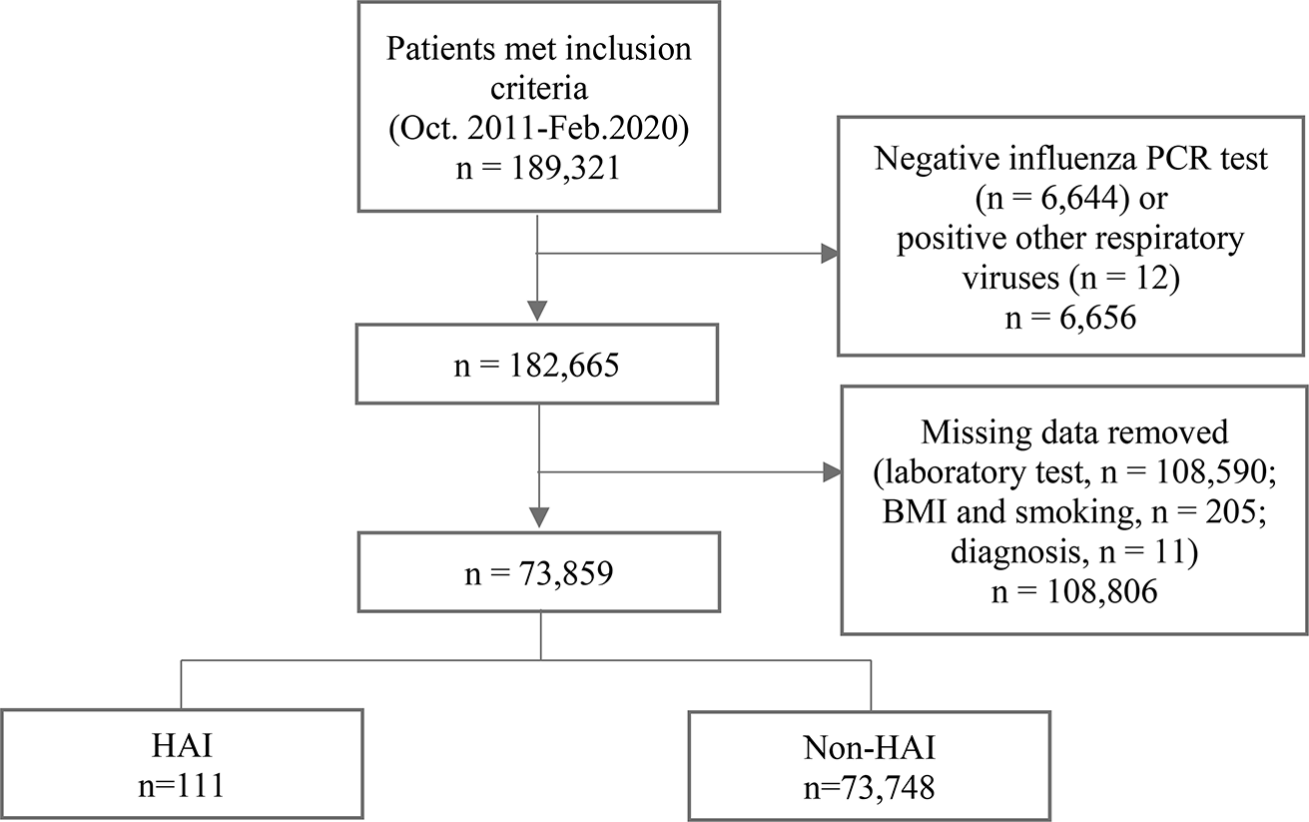
Study sample selection. HAI Hospital-acquired influenza, PCR Polymerase chain reaction, BMI Body mass index

The sample consisted of patients aged 19 years and older, who had stayed in the general adult wards for more than four days. Patients solely diagnosed with influenza and showing a positive polymerase chain reaction (PCR) test within four days of admission were excluded because of their classification as cases of CAI infections. Patients who had undergone surgery during admission were also excluded. In total, 189,321 patients were included in the study, comprising 117 HAI patients and 182,204 non-HAI patients (Fig. 1). Patients with negative PCR results were typically categorized as non-HAI cases. However, given that these individuals underwent testing because they exhibited symptoms and considering the inherent non-100% accuracy of the test, it is possible that some of them could indeed be HAI cases. To mitigate this uncertainty, patients were excluded from the analysis to prevent any potentially skewed impact on the training of the predictive model.

### Outcome and predictor variables

The outcome variable was the presence of HAI. HAI patients were defined as those with a positive result from an influenza A or B PCR test conducted more than four days after admission. Patients who did not undergo PCR were categorized as non-HAI.

The predictor variables were chosen based on an extensive literature review, considering the factors influencing influenza. General characteristics included sex [23], age [1, 3, 8, 23–30], body mass index (BMI) [11], pregnancy status [3, 11], smoking history (past or present) [31], immunosuppression status [1–4, 8, 23, 32], and corticosteroid use [33] (Appendix Table A.1). Comorbidities were ascribed if patients had received diagnoses of diabetes [2, 8, 9], obesity [11], heart disease [2, 8, 11, 23], liver disease [9, 11], renal disease [2, 8, 11, 32], hematologic disease [3, 11, 23], malignancy [1, 4, 9], organ transplantation [1], asthma [11], or chronic obstructive pulmonary disease (COPD) [8, 9, 11, 32] before the index date. This study applied the method of means and changes from previous values [34] to transform vital signs, including body temperature (BT), heart rate (HR), respiration rate (RR), systolic blood pressure (SBP), and diastolic blood pressure (DBP) [35].

Laboratory results [23] and haematological inflammatory parameters, specifically the neutrophil-to-lymphocyte ratio (NLR), platelet-to-neutrophil ratio (PNR), and platelet-to-lymphocyte ratio (PLR) [36], were included. The radiological results consisted of selected chest X-ray findings [23]. Patient rooms and units were included as factors because the type of hospital room [24, 37] and sharing a room or unit with an influenza patient [9, 38] are risk factors for HAI infection.

The observation period for each patient spanned four days before the index date, considering the incubation period of influenza [39]. The index date corresponded to the PCR test date [32, 35], except for patients who did not undergo PCR testing, for whom the index date was established on the fifth day after admission.

### Data preparation

Among these were no laboratory results for 108,590 patients, while 205 had missing smoking or BMI information, and 11 had no diagnostic information. Finally, 108,806 patients were excluded (Fig. 1). This resulted in the remaining 73,859 patients, of whom 111 exhibited HAI. In cases where certain laboratory test results were missing, the following approach was adopted despite the presence of other results. Due to an absence rate of 80.8% among the patients, the direct bilirubin variable was removed. For other laboratory results, the missing rates were less than 5%, including calcium (4.6%), total bilirubin (3.7%), alanine transaminase (ALT; 2%), albumin (1.1%), aspartate transaminase (AST; 0.9%), blood urea nitrogen (BUN; 0.4%), creatinine (0.3%), and CO2 (0.02%). Consequently, imputation was employed to address missing data for laboratory test variables. The absence of laboratory test results indicated that the attending physician did not consider the test necessary for the patient; therefore, missing laboratory test results were not considered abnormal [40]. Continuous laboratory variables were imputed using the median values within the normal range.

Of the 73,859 patients included in this study, only 111 (0.15%) were diagnosed with HAI, which resulted in an unbalanced dataset. Imbalanced classes are common in real-world healthcare data and can diminish the predictive efficacy of models [41]. To address this issue, a synthetic minority oversampling technique (SMOTE) was employed, which involves generating new and reasonably accurate data based on existing minority cases [41]. SMOTE generates data by computing the Euclidean distance between any two randomly selected k-nearest neighbours (KNN) from two minority samples and creating new data points along the line connecting them [41].

### Feature selection

Feature selection is a prevalent technique in forecasting, pattern recognition, and classification modelling, designed to reduce the dimensionality and complexity of datasets by eliminating irrelevant and redundant features [42]. Various methods, including Information GainRatio Attribute Evaluation (GA), Forward Elimination, Backward Elimination, and One Rule Attribute Evaluation (ORAE), have been proposed for selecting pertinent features in predictive modelling [43]. In this study, we employed RFECV (Recursive Feature Elimination with Cross-Validation), a form of Backward Elimination, utilising a random forest classifier as the estimator with accuracy as the scoring metric, for feature selection. As a result, the following 36 variables were retained, encompassing features such as age, sex, BMI, malignancy, BT, HR, RR, SBP, DBP, red blood cell (RBC), haemoglobin (Hb), white blood cell (WBC), platelet, haematocrit, RDW, delta neutrophil index (DNI), neutrophil, lymphocyte, NLR, PNR, PLR, sodium, potassium, chloride (Cl), CO2, calcium, albumin, total bilirubin, BUN, creatinine, ALT, AST, normal chest X-ray, abnormal chest X-ray, multi-occupancy room, and double room (variables marked with an asterisk in Appendix Table A.1).

### Model development

After processing the raw data, 53 variables were categorized into seven groups (see Appendix Table A.1). Descriptive and univariate analyses were performed to determine the characteristics and factors associated with HAI. Chi-square and t-tests were used to analysed categorical and continuous variables, respectively.

To develop prediction models for HAI, a combination of ML classification methods, including Random Forest (RF), Extreme Gradient Boosting (XGB), Artificial Neural Networks (ANN), and Logistic Regression (LR), was employed with the selected 36 variables. LR, widely utilized for predicting patient outcomes, such as mortality or disease onset, was juxtaposed with ML methods in healthcare data analysis studies [16]. RF is an ensemble model of decision trees that amalgamates multiple weak classifier models into a robust model that outperforms individual components [44]. Decision-tree algorithms can be sensitive to minor cases in datasets; however, RF mitigates this by aggregating the outcomes of various decision trees [45]. Despite their longer training times, straightforward ensemble models exhibit noteworthy performance [44, 46]. XGB builds on the gradient boosting model, known for its reliability but has a prolonged training period. XGB considerably reduces this training duration, rendering it one of the most advanced supervised ML algorithms and faster than other ensemble classifiers [44]. ANNs possess significant predictive capability among classification algorithms and are extensively employed. The transparency and interpretability of models hold significance within healthcare [16] to explicate the rationale underlying outcomes. Despite their limitations in interpretability, ANNs have demonstrated robust predictive properties.

Five-fold grid search cross-validation (GSCV) was performed on the training set. GSCV identifies the optimal combination of hyperparameters that enhances model performance while preventing overfitting [44]. The optimized hyperparameters for each ML model examined in this study were as follows. The RF model featured a maximum depth of 20 m, a minimum of two sample splits, and 100 n estimators. The XGB model had a maximum depth of 5, a learning rate of 0.2, a subsample of 0.75, and 10 n estimators. The ANN model comprised 50 and 100 activation-rectified linear units, a hidden layer size of 50, a learning rate of 0.005, and an Adam solver.

### Model evaluation

It is imperative that the models not be trained or evaluated using the same dataset to ascertain their accuracy [47]. In this study, 80% of the dataset was randomly assigned to the training set, and the remaining 20% was assigned to the test set. No variables showed significant differences between the training and test sets (see Appendix Table A.2). The assessment of the discriminatory ability of a classification model involves metrics such as accuracy, sensitivity, specificity, and area under the receiver operating characteristic curve (AUC) [48]. In this study, particular emphasis was placed on the AUC and the number of false negatives (FN). AUC, the most commonly used metric for evaluating prediction models and FN count, is crucial in healthcare as it signifies untreated patients potentially spreading the virus and is deemed paramount. In addition, SHAP (SHapley Additive exPlanations) was employed to assess feature importance by utilizing Shapley values [49]. This methodology considers contributions across all possible combinations for fair attribution, accommodating feature interactions and enabling a more accurate evaluation of individual feature importance [49]. SHAP is versatile, applicable to diverse machine learning models, including regression, classification, and ensemble models. Visualized through a dot plot, the results depict Shapley values for each feature, offering an intuitive understanding of their impact on model predictions. Positive values indicate contributions that increase predictions, while negative values suggest contributions that decrease predictions. This analysis provides clear insights into the most influential features, contributing valuable information for a quantitative interpretation of the model’s feature importance [49].

Data analysis was performed using SQL Server Management Studio v18.10 (Microsoft, Seattle, US) and Python 3.5. SQL was used to integrate, preprocess, and transform the data. Python was used for the univariate analyses and ML.

### Ethical considerations

This study was approved by the Yonsei University Health System Institutional Review Board (IRB No. 4-2021-1252) and Data Review Board (DRB No. 2,021,300,331). After obtaining approval, the data were extracted and anonymized by authorized personnel from the hospital’s records management department before being sent to the researcher.

## Results

### Characteristics of HAI patients

Table 1 presents an overview of the characteristics of the HAI patients. Patients with HAI exhibited an average LoS of 12.5 days (SD = 10.9 days) at the time of PCR testing. Their total LoS significantly exceeded that of the non-HAI patients (*p* < 0.001). Patients with HAI were also older (*p* < 0.001) and had higher immunosuppression and corticosteroid use rates (both *p* < 0.001). Significant differences were observed in the prevalence of diabetes (*p* < 0.001), heart disease (*p* < 0.001), renal disease (*p* < 0.001), haematological disease (*p* = 0.037), asthma (*p* < 0.001), and COPD (*p* < 0.001). Additionally, patients with HAI exhibited greater variations in BT, HR, SBP, and DBP than non-HAI patients.

In terms of laboratory results, HAI patients had lower RBC counts, Hb levels, platelet counts, haematocrit levels, and lymphocyte counts (all *p* < 0.001). In contrast, RDW, DNI, and PLR were higher in HAI patients (*p* = 0.007, *p* = 0.02, and *p* = 0.04, respectively). Sodium, potassium, Cl, calcium, albumin, and total bilirubin levels were lower in patients with HAI. Conversely, HAI patients had higher BUN levels (*p* = 0.024). More HAI patients showed abnormal chest X-ray findings (*p* < 0.001) and had higher rates of co-location with influenza patients in the same room, unit, and double room (all *p* < 0.001) than non-HAI patients.

**Table 1:** Characteristics of HAI and non-HAI patients

Variable	Total (*n* = 73,859)	Non-HAI (*n* = 73,748)	HAI (*n* = 111)	t or χ^2^		*p*-value		
General characteristics								
LoS at PCR testing, days, mean (SD)	-	-	12.5 (10.9)	-		-		
Total LoS, days, mean (SD)	12.5 (15.3)	12.5 (15.3)	27.0 (23.1)	-6.641		< 0.001	^***^	
Age, years, mean (SD)	58.7 (16.1)	58.7 (16.1)	68.8 (13.0)	-8.220		< 0.001	^***^	
Sex, male, n (%)	40,588 (55.0)	40,529 (55.0)	59 (53.2)	0.082		0.775		
BMI, mean (SD)	23.0 (3.6)	23.0 (3.6)	22.9 (4.5)	0.332		0.740		
Pregnant, n (%)	716 (1.0)	716 (1.0)	0 (0.0)	0.312		0.577		
Ex-smoker, n (%)	15,161 (20.5)	15,135 (20.5)	26 (23.4)	0.408		0.523		
Current smoker, n (%)	9,928 (13.4)	9919 (13.4)	9 (8.1)	2.278		0.131		
Immunosuppressed, n (%)	19,543 (26.5)	19,495 (26.4)	48 (43.2)	15.240		< 0.001	^***^	
Corticosteroid use, n (%)	25,035 (33.9)	24,972 (33.9)	63 (56.8)	24.918		< 0.001	^***^	
Comorbidities, n (%)								
Diabetes	4,653 (6.3)	4,635 (6.3)	18 (16.2)	16.875		< 0.001	^***^	
Obesity	35 (0.0)	35 (0.0)	0 (0.0)	0.000		1		
Heart disease	8,207 (11.1)	8,176 (11.1)	31 (27.9)	30.146		< 0.001	^***^	
Liver disease	4,825 (6.5)	4,816 (6.5)	9 (8.1)	0.230		0.631		
Renal disease	9,104 (12.3)	9,074 (12.3)	30 (27.0)	20.890		< 0.001	^***^	
Hematologic disease	4,733 (6.4)	4,720 (6.4)	13 (11.7)	4.366		0.037	^*^	
Malignancy	37,214 (50.4)	37,166 (50.4)	48 (43.2)	1.991		0.158		
Organ transplantation	3,600 (4.9)	3,596 (4.9)	4 (3.6)	0.161		0.688		
Asthma	926 (1.3)	915 (1.2)	11 (9.9)	60.462		< 0.001	^***^	
COPD	1,095 (1.5)	1,081 (1.5)	14 (12.6)	86.809		< 0.001	^***^	
Vital signs, mean (SD)								
Largest variation for BT, ºC	0.8 (0.4)	0.8 (0.4)	1.0 (0.4)	-6.117	< 0.001		^***^	
Largest variation for HR, beats/m	16.4 (10.1)	16.4 (10.1)	19.3 (10.5)	-3.082	0.002		^**^	
Largest variation for RR, beats/m	2.4 (4.3)	2.4 (4.3)	2.4 (2.8)	-0.033	0.741			
Largest variation for SBP (mmHg)	23.1 (12.6)	23.1 (12.6)	27.4 (12.5)	-3.610	< 0.001		^***^	
Largest variation for DBP (mmHg)	16.5 (8.4)	16.5 (8.4)	19.1 (8.5)	-3.340	< 0.001		^***^	
Laboratory test results, mean (SD)								
RBC count (10^3^/Μl)	3.7 (0.7)	3.7 (0.7)	3.3 (0.7)	5.431	< 0.001		^***^	
Hb (g/Dl)	11.3 (2.0)	11.3 (2.0)	10.2 (1.9)	5.723	< 0.001		^***^	
WBC count (10^3^/Μl)	7.6 (4.5)	7.6 (4.5)	6.9 (4.6)	1.571	0.116			
Platelet count (10^3^/Μl)	221.0 (108.6)	221.0 (108.6)	184.8 (102.5)	3.509	< 0.001		^***^	
Haematocrit (%)	33.7 (5.8)	33.7 (5.8)	30.5 (5.8)	5.864	< 0.001		^***^	
RDW (%)	14.6 (2.2)	14.6 (2.2)	15.2 (2.3)	-2.703	0.007		^**^	
DNI (%)	1.4 (3.2)	1.4 (3.2)	1.9 (2.0)	-2.366	0.020		^*^	
Neutrophil count (10^3^/Μl)	5.5 (4.1)	5.5 (4.1)	5.0 (3.4)	1.624	0.107			
Lymphocyte count (10^3^/Μl)	1.3 (0.7)	1.3 (0.7)	0.9 (0.6)	6.711	< 0.001		^***^	
NLR	7.1 (48.2)	7.1 (48.2)	8.7 (9.3)	-1.797	0.075			
PNR	58.8 (145.5)	58.8 (144.7)	102.6 (427.0)	-1.080	0.283			
PLR	237.8 (356.5)	237.6 (356.2)	329.8 (468.0)	-2.073	0.040		^*^	
Sodium (mmol/L)	138.9 (3.9)	138.9 (3.9)	137.8 (4.5)	2.679	0.009		^***^	
Potassium (mmol/L)	4.0 (0.5)	4.0 (0.5)	3.8 (0.5)	4.370		< 0.001	^***^	
Cl (mmol/L)	102.7 (4.4)	102.8 (4.4)	101.1 (5.0)	3.420		< 0.001	^***^	
CO2 (mmol/L)	23.8 (3.3)	23.8 (3.3)	23.6 (3.6)	0.729		0.466		
Calcium (mmol/L)	8.5 (0.7)	8.5 (0.7)	8.2 (0.8)	4.236		< 0.001	^***^	
Albumin (mmol/L)	3.4 (0.6)	3.4 (0.6)	3.0 (0.6)	5.912		< 0.001	^***^	
Total bilirubin (mmol/L)	1.0 (1.9)	1.0 (1.9)	0.8 (1.0)	2.227		0.028	^*^	
BUN (mg/Dl)	17.3 (13.5)	17.3 (13.5)	21.0 (16.8)	-2.296		0.024	^*^	
Creatinine (mg/Dl)	1.1 (1.3)	1.1 (1.3)	1.3 (1.3)	-1.540		0.124		
ALT (mmol/L)	36 (98.9)	36 (99)	33.8 (55.6)	0.398		0.692		
AST (mmol/L)	41.4 (163.4)	41.4 (163.4)	45.6 (101.8)	-0.423		0.673		
Radiology test result, n (%)								
Chest X-ray, Normal	25,121 (34)	25,111 (34)	10 (9.0)					
Abnormal	42,027 (56.9)	41,926 (56.9)	101 (91.0)					
None	6,711 (9.1)	6,711 (9.1)	0 (0)	53.237		< 0.001	^***^	
Room status, n (%)								
Same room	1,542 (2.1)	1,526 (2.1)	16 (14.4)	76.703		< 0.001	^***^	
Same unit	9,146 (12.4)	9,095 (12.3)	51 (45.9)	112.340		< 0.001	^***^	
Multi-occupancy room	64,858 (87.8)	64,763 (87.8)	95 (85.6)	0.328		0.567		
Double room	35,534 (48.1)	35,508 (48.1)	26 (23.4)	26.158		< 0.001	^***^	

^*^ p-value ≤ 0.05, ^**^p-value ≤ 0.01, ^***^p-value ≤ 0.001

LoS Length of stay, PCR Polymerase chain reaction, COPD Chronic obstructive pulmonary disease, BT Body temperature, HR Heart rate, RR Respiration rate, SBP Systolic blood pressure, DBP Diastolic blood pressure, RBC Red blood cell, Hb Haemoglobin, WBC White blood cell, RDW Red blood cell distribution width, DNI Delta neutrophil index, NLR Neutrophil-to-lymphocyte ratio, PNR Platelet-to-neutrophil ratio, PLR Platelet-to-lymphocyte ratio, Cl Chloride, BUN Blood urea nitrogen, ALT Alanine transaminase, AST Aspartate transaminase

### Prediction model development

**Fig. 2:**
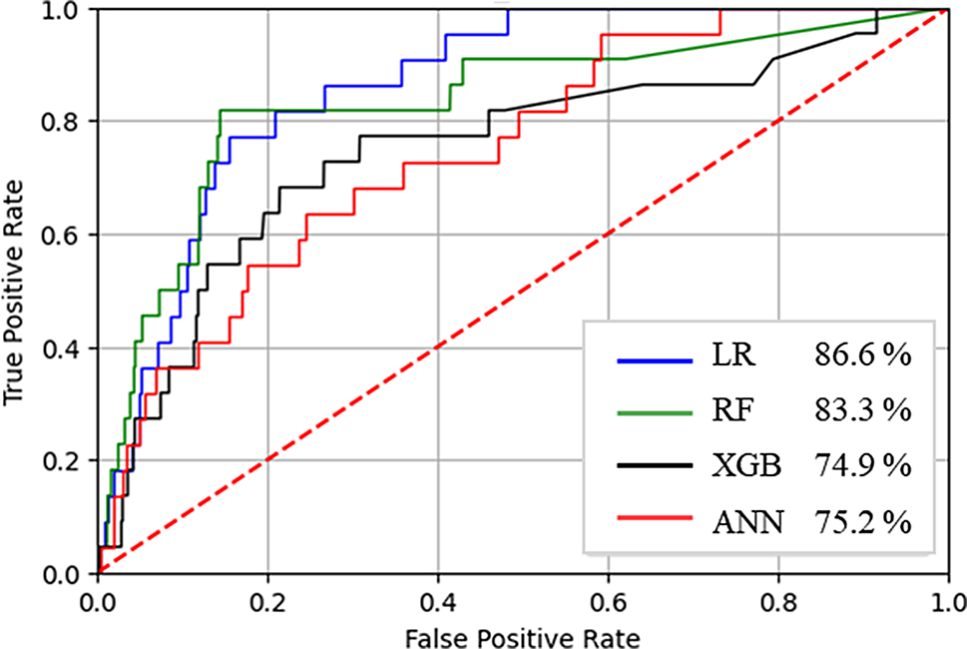
ROC curves and AUCs. LR Logistic Regression, RF Random Forest, XGB Extreme Gradient Boosting, ANN Artificial Neural Network

Prediction models were developed using the LR, RF, XGB, and ANN ML techniques. The LR model had the highest AUC (86.6%), followed by RF (83.3%), ANN (74.9%), and XGB (75.2%) (Table 2). In addition, the RF model exhibited the lowest number of FN at four, followed by LR (five), ANN (six), and XGB (eight). A visual representation of the receiver operating characteristics (ROC) curves and AUC values for all models is presented in Fig. 2.

**Table 2:** Model evaluation results

Model	AUC (%)	Sensitivity	Specificity	Accuracy	F_1_ Score	TP (*n*)	TN (*n*)	FP (*n*)	FN (*n*)
LR	86.6	0.77	0.79	79.1	1.1	17	11,671	3,079	5
RF	83.3	0.82	0.82	82.2.	1.3	18	12,119	2,631	4
XGB	74.9	0.73	0.73	72.9	0.8	16	10,747	4,003	6
ANN	75.2	0.64	0.70	70.0	0.6	14	10,320	4,430	8

LR Logistic Regression, RF Random Forest, XGB Extreme Gradient Boosting, ANN Artificial Neural Network, AUC Area under the receiver operating characteristics curve, TP True positive, TN True negative, FP False positive, FN False negative

**Fig. 3:**
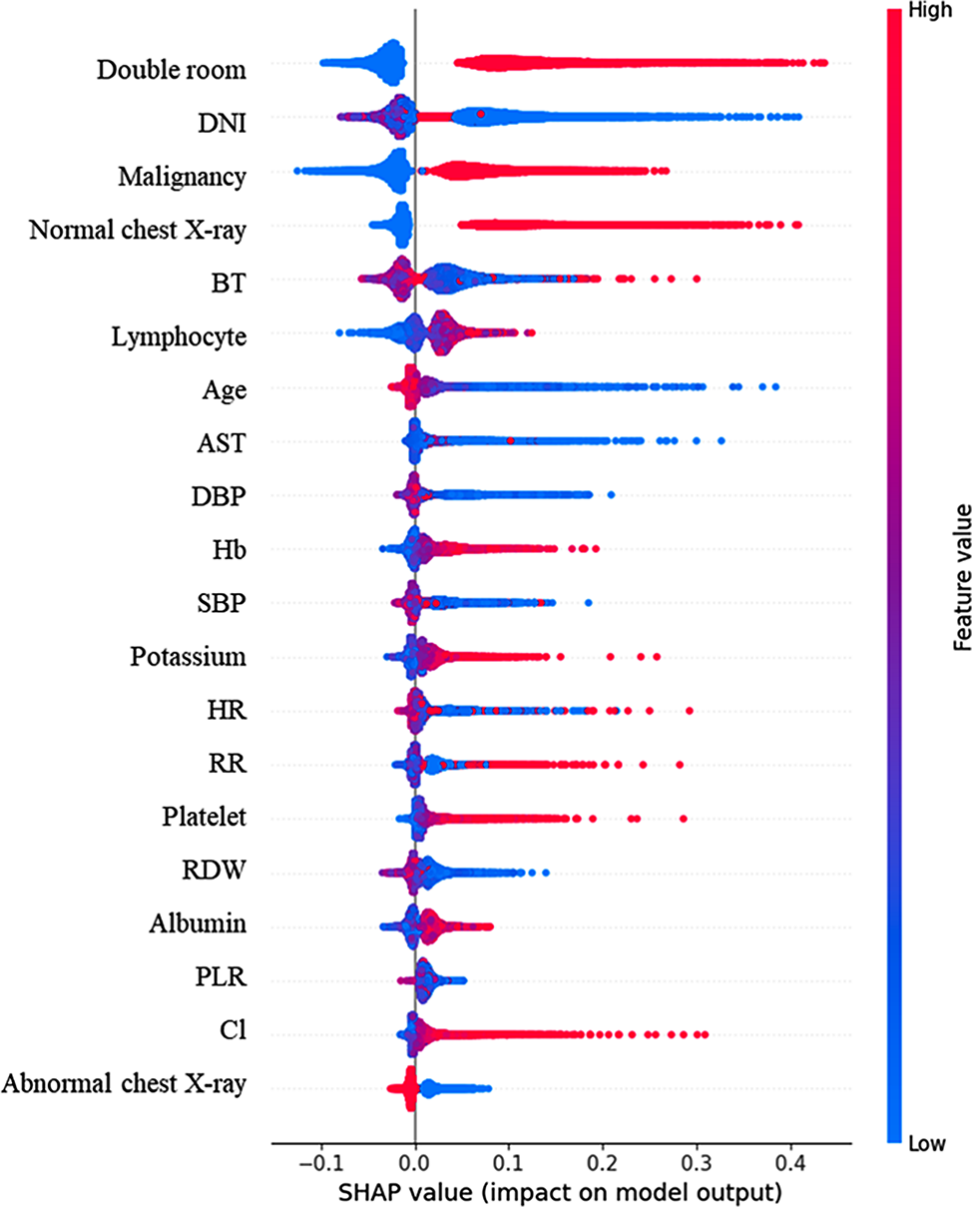
Results of the analysis on feature importance using RF. DNI Delta neutrophil index, BT Body temperature, AST Aspartate transaminase, DBP Diastolic blood pressure, Hb Haemoglobin, SBP Systolic blood pressure, HR Heart rate, RR Respiration rate, RDW Red blood cell distribution width, PLR Platelet-to-lymphocyte ratio, Cl Chloride

The major results of the feature importance analysis using RF are shown in Fig. 3. The results of the feature importance analysis for LR, XGB and ANN are presented in Figures A.1, A.2, and A.3 in the Appendix. Occupying a double room ranked the highest among the significant factors, followed by the DNI, malignancy, chest X-ray findings, and BT. Notably, all five vital sign attributes (BT, DBP, SBP, HR and RR) and ten laboratory variables (DNI, lymphocyte, AST, Hb, potassium, platelet, RDW, albumin, PLR, and Cl) were among the top 20 most influential factors.

## Discussion

### Characteristics of HAI patients

In this study, patients with HAI underwent PCR testing on average 12.5 days after admission, which aligned with the findings of Bischoff et al. [35] at 12.4 days. This implies an elevated vulnerability to HAI infection with prolonged hospital stay. In addition, HAI patients had an average LoS that exceeded that of non-HAI patients by 14.5 days. Similarly, studies have reported longer hospital stays for HAI patients than non-HAI patients [23] and patients [2–4, 35].

Most studies concentrated on contrasting HAI patients with CAI rather than non-HAI patients. Nevertheless, the outcomes of the present study align closely with the findings of those investigations. HAI patients were, on average, older than non-HAI patients [1, 3, 8, 35]. Furthermore, patients with HAI demonstrate an increased likelihood of immunosuppression [1–4, 8, 23, 32, 50], diabetes [8, 9], heart disease [2, 8, 23, 32], renal disease [2, 8, 32], hematologic disease [3], and COPD [32].

This study revealed that patients with HAI displayed higher variations from the preceding 24-hour average in BT, HR, SBP, and DBP than non-HAI. Notably, Bischoff et al. [35], who compared HAI and CAI patients, found no similar distinctions. This disparity can be attributed to using raw values in their study. Conversely, Churpek et al. [34] emphasized the importance of variations in vital signs rather than their absolute values. Considering the limited exploration of the connection between vital signs and HAI, further investigation is warranted.

Regarding haematological parameters, HAI patients exhibited lower RBC, Hb, platelet, haematocrit, and lymphocyte counts, while RDW, DNI, and PLR were elevated compared with non-HAI patients. These findings align with those of Yang et al. [23], particularly in the case of lymphocyte counts, although disparities were observed in Hb and platelet counts. Our findings for RBC, Hb, platelets, lymphocytes, RDW, and PLR closely resembled those of Han et al.’s investigation [36], which involved comparing influenza patients and healthy individuals.

Han et al. [36] reported reduced platelet levels in an influenza infection group compared with healthy and negative control groups. The negative control group experienced respiratory symptoms but tested negative for influenza or bacterial infection. Interestingly, the platelet counts in the influenza group returned to normal upon recovery. In addition to their role in coagulation, platelets are recognized as significant inflammatory cells [51]. Influenza viruses can increase platelet activation [51], decreasing platelet counts [36]. Consequently, a diminished platelet count could serve as a distinguishing factor for influenza infection from other infections [36].

Other haematological inflammatory markers, such as neutrophil and WBC counts, were higher in influenza patients than in healthy individuals; however, these counts were lower than those observed in patients infected with bacteria [36]. These consistent findings correspond with our non-significant results, which parallel the findings of Yang et al. [23]. This suggests that neutrophil and WBC counts may exhibit greater variability than platelet counts between individuals with and without influenza infections in contrast to platelet counts [36]. Moreover, the PLR yielded a significant result among the various blood cell indices, while the PNR and NLR did not exhibit significance in our study. Given that both PNR and NLR involve neutrophil counts, which were also non-significant, further research is warranted to explore the diverse associations of haematological parameters with patient conditions.

In this study, all HAI patients underwent chest X-rays, compared to 90.9% of the non-HAI patients. Among the HAI patients, 91% exhibited abnormal findings, whereas only 56.9% of the non-HAI patients did so. Similarly, Yang et al. [23] noted an elevated incidence of pleural effusion in chest X-ray results of HAI patients. This underscores the increased susceptibility of individuals with anomalous chest X-ray findings to HAI.

A higher proportion of HAI patients occupied rooms or units shared with influenza patients than non-HAI patients. Furthermore, HAI was more prevalent in double-occupied rooms, with no difference observed in multi-occupancy rooms. Multi-occupancy rooms are more congested than double-occupied rooms, increasing the presence of occupants, caregivers, visitors, and the risk of influenza infection. However, patients in double rooms consistently remained near potentially infected individuals, whereas those in multi-occupancy rooms maintained a greater distance. Although the recommended 1.8-meter distance [11] from influenza patients was not met in either room type, patients in the double room could be more susceptible to droplet exposure. Frequent door openings in multi-occupancy rooms are likely to enhance ventilation, particularly during months when windows are unlikely to open, a trend indicated by influenza peak seasons. Wong et al. [52] and Xiao et al. [53] emphasized the importance of aerosol transmission and its critical role in influenza transmission. This study highlights the importance of aerosols and clarifies why influenza infection was associated with a stay in double rooms, whereas a stay in multi-occupancy rooms was not.

Identifying disparities in the characteristics of HAI and non-HAI patients presents a challenge because of their shared severe medical conditions that necessitate hospitalization. Nonetheless, this study successfully identified the differentiating characteristics between the two groups. Hospitals can employ these insights to formulate infection prevention strategies to mitigate influenza transmission in healthcare facilities.

### HAI prediction model

This study represents a pioneering effort to develop a HAI prediction model by applying ML techniques. Both the LR (86.6%) and RF (83.3%) models demonstrated AUC exceeding 80%, with RF yielding the lowest FN count (four), followed by LR (five). Consequently, the RF model was the most suitable candidate for clinical implementation.

Notably, the most pivotal predictor of HAI was the occupation of double room. As discussed, patients residing in double rooms may face heightened susceptibility to aerosol-borne infections owing to their proximity to potential sources of infection and constrained ventilation in such settings. The second most influential feature was the DNI, which assumes special significance during the initial stages of infection. Overproduction of cytokines and chemokines during this period obstructs the migration of neutrophils to the infection site, releasing immature neutrophils into the bloodstream, a phenomenon termed left-shifting [54]. DNI, which represents the proportion of immature granulocytes among neutrophils in the peripheral circulation, increases in left-shifting cases [55]. The DNI has demonstrated superior predictive capacity for infections and prognosis compared to WBC, C-reactive protein, or neutrophil counts [56]. As the DNI effectively discriminates between low-grade community-acquired pneumonia and common colds [56], its significance in predicting HAI was reaffirmed in this study.

Patients with HAI showed more variation in BT, HR, SBP, and DBP than non-HAI patients. All five vital signs are ranked within the top 14 predictors. This highlights the potential of predicting HAI infections. Vital signs are commonly used to predict clinical deterioration [34] and diseases such as acute GVHD [12] and sepsis [16]. This study reinforces the importance of vital signs in predicting HAI.

This study underscores the importance of vital signs, diverse laboratory results, and chest X-ray findings in distinguishing between HAI and non-HAI patients for predicting HAI infections. Notably, sex, smoking status, immunosuppression, room allocation, and comorbidities exhibited relatively lower predictive values than vital signs, laboratory outcomes, and chest X-ray result, as indicated by the feature importance analysis. This suggests that the latter group reflects immediate patient conditions, whereas the demographic and medical history variables may not have the same predictive power. Additionally, these variables were observed during the incubation period, implying that changes in vital signs, laboratory findings, and chest X-ray results could manifest even before the onset of typical influenza-like symptoms in patients with influenza. This highlights the potential of immediate patient conditions during the incubation period to offer predictive insights before the emergence of typical influenza-like symptoms.

## Limitations

This study had several limitations. First, its single-centre nature at a tertiary teaching hospital raises concerns about generalizability, necessitating broader hospital settings for validation. The imbalanced dataset proportions (HAI patients at 0.15%) were addressed using the SMOTE method. Reliance on EMR from a single centre may not fully represent patients’ medical histories, focusing on selected inpatient visits and omitting influenza vaccination and home medication data. This retrospective design hindered the inclusion of healthcare provider, caregiver, and visitor information in the context of influenza transmission. This study explored only four ML techniques; however, broader methodological considerations could enhance its applicability.

## Conclusion

This study revealed the pivotal attributes, medical indicators, subtle changes in vital signs, and laboratory outcomes of patients with HAI. The critical role of effective ventilation in preventing hospital-acquired influenza has been underscored. These findings will enrich infection prevention strategies in healthcare settings. Furthermore, predictive models offer prospects for pre-emptive interventions to curb influenza dissemination within hospital settings.

### Electronic supplementary material

Below is the link to the electronic supplementary material.

Supplementary Material 1

## Data Availability

The data for this article were provided by the Yonsei University Health System with permission, subsequent to Institutional Review Board approval. Requests for data access can be made to the corresponding author, subject to permission from the Yonsei University Health System.

## Abbreviations

HAI: Hospital-acquired influenza
CAI: Community-acquired influenza
PCR: Polymerase chain reaction
BMI: Body mass index
COPD: Chronic obstructive pulmonary disease
BT: Body temperature
HR: Heart rate
RR: Respiration rate
SBP: Systolic blood pressure
DBP: Diastolic blood pressure
NLR: Neutrophil-to-lymphocyte ratio
PNR: Platelet-to-neutrophil ratio
PLR: Platelet-to-lymphocyte ratio
ALT: Alanine transaminase
AST: Aspartate transaminase
BUN: Blood urea nitrogen
SMOTE: Synthetic minority oversampling technique
KNN: k-nearest neighbours
DNI: Delta neutrophil index
Hb: Haemoglobin
WBC: White blood cell
RF: Random Forest
XGB: Extreme Gradient Boosting
ANN: Artificial Neural Network
LR: Logistic Regression
GSCV: Grid search cross-validation
AUC: Area under the receiver operating characteristic curve
RBC: Red blood cell
RDW: Red blood cell distribution width
Cl: Chloride
TP: True positive
TN: True negative
FP: False positive
FN: False negative
