# Supplementary material for: Prediction of hospital-acquired influenza using machine learning algorithms: a comparative study
Source: BMC Infect Dis. 2024 May 2;24:466. doi: 10.1186/s12879-024-09358-1 (PMC11067145; doi:10.1186/s12879-024-09358-1)
Supplement: Supplementary file 1 — Supplementary Material 1 [file 12879_2024_9358_MOESM1_ESM.docx]

## Appendix

| Table A.1. Study variables | | | |
| --- | --- | --- | --- |
| Category | Variables | Type  /Level | Description |
| Seasonal influenza | HAI | Y/N | A positive PCR test for influenza A or B more than four days after admission |
| General characteristics | |  |  |
|  | Age^*^ | Number | Age on admission |
|  | Sex^*^ | M/F |  |
|  | BMI^*^ | Number |  |
|  | Pregnancy status | Y/N |  |
|  | Ex-smoker status | Y/N |  |
|  | Current smoker status | Y/N |  |
|  | Immuno-suppressed | Y/N | Immunosuppressant (10 mg or more of prednisolone‐equivalent steroids, monoclonal antibodies, antimetabolite drugs, or T‐cell inhibitors [22]) administered during this admission |
|  | Corticosteroid use | Y/N | Administered during this admission |
| Comorbidities | |  | Based on ICD 10 codes from admission to the index date |
|  | Diabetes | Y/N | E10, E11, E13 |
|  | Obesity | Y/N | E66.9 |
|  | Heart disease | Y/N | I05–I09, I20–I25, I27, I30–I52 |
|  | Liver disease | Y/N | K70–K77 |
|  | Renal disease | Y/N | N00–N08, N10–N16, N17–N19,  N25–N29 |
|  | Hematologic disease | Y/N | D50–D53, D55–D59, D60 –D69, D70–D77 |
|  | Malignancy^*^ | Y/N | C00–C97 |
|  | Organ transplantation | Y/N | Z94 |
|  | Asthma | Y/N | J45, J46 |
|  | COPD | Y/N | J44 |
| Vital signs |  |  | Largest differences between the current and the average of the three preceding values |
|  | BT^*^ | Number |  |
|  | HR^*^ | Number |  |
|  | RR^*^ | Number |  |
|  | SBP^*^ | Number |  |
|  | DBP^*^ | Number |  |
| Laboratory results | | | Latest value during the observation period |
|  | RBC count^*^ | Number |  |
|  | Haemoglobin^*^ | Number |  |
|  | WBC count^*^ | Number |  |
|  | Platelet count^*^ | Number |  |
|  | Haematocrits^*^ | Number |  |
|  | RDW^*^ | Number |  |
|  | DNI^*^ | Number |  |
|  | Neutrophil count^*^ | Number |  |
|  | Lymphocyte count^*^ | Number |  |
|  | NLR^*^ | Number | The neutrophil count divided by the lymphocyte count |
|  | PNR^*^ | Number | The platelet count divided by the neutrophil count |
|  | PLR^*^ | Number | The platelet count divided by the lymphocyte count |
|  | Sodium^*^ | Number |  |
|  | Potassium^*^ | Number |  |
|  | Cl^*^ | Number |  |
|  | CO2^*^ | Number |  |
|  | Calcium^*^ | Number |  |
|  | Albumin^*^ | Number |  |
|  | Total bilirubin^*^ | Number |  |
|  | Direct bilirubin | Number | Removed, missing rate: 80.8% |
|  | BUN^*^ | Number |  |
|  | Creatinine^*^ | Number |  |
|  | ALT^*^ | Number |  |
|  | AST^*^ | Number |  |
| Radiology result | |  |  |
|  | Chest X-ray^*^ | (0/1/9) | Normal/Abnormal/None |
| Room information | |  |  |
|  | Same room | Y/N | Share a room with an influenza patient |
|  | Same unit | Y/N | Stay a same unit with an influenza patient |
|  | Multi-occupancy room^*^ | Y/N | Rooms capable of housing more than two patients |
|  | Double room^*^ | Y/N |  |

* Selected features to develop prediction models

*HAI* Hospital-acquired influenza, *PCR* Polymerase chain reaction, *BMI* Body mass index,

*ICD* International Classification of Diseases, *COPD* Chronic obstructive pulmonary disease, *BT* Body temperature, *HR* Heart rate, *RR* Respiration rate, *SBP* Systolic blood pressure, *DBP* Diastolic blood pressure,

*RBC* Red blood cell, *WBC* White blood cell, *RDW* Red blood cell distribution width, *DNI* Delta neutrophil index, *NLR* Neutrophil-to-lymphocyte ratio, *PNR* Platelet-to-neutrophil ratio, *PLR* Platelet-to-lymphocyte ratio,

*Cl* Chloride, *BUN* Blood urea nitrogen, *ALT* Alanine transaminase, *AST* Aspartate transaminase

| Table A.2. The distribution between training set and test set | | | | | | | | | | | |
| --- | --- | --- | --- | --- | --- | --- | --- | --- | --- | --- | --- |
| Variable | | Total  (n = 73,859) | | Training  (n = 59,087) | | Test  (n = 14,772) | | t or χ^2^ | p-value |  | |
| General characteristics | |  | |  | |  | |  |  |  | |
| Age,  years, mean (SD) | | 58.7 (16.1) | | 58.7 (16.1) | | 58.6 (16.0) | | 0.846 | 0.396 |  | |
| Sex, male, n (%) | | 40,588 (55.0) | | 32,558 (55.1) | | 8,030 (54.5) | | 2.600 | 0.107 |  | |
| BMI, mean (SD) | | 23.0 (3.6) | | 23.0 (3.6) | | 23.0 (3.6) | | 0.766 | 0.444 |  | |
| Ex-smoker, n (%) | | 15,161 (20.5) | | 12,150 (20.6) | | 3,011 (20.4) | | 0.223 | 0.637 |  | |
| Current smoker,  n (%) | | 9,928 (13.4) | | 7,943 (13.4) | | 1,985 (13.4) | | 0.000 | 0.997 |  | |
| Immunosuppressed,  n (%) | | 19,543 (26.5) | | 15,619 (26.4) | | 3,924 (26.6) | | 0.096 | 0.757 |  | |
| Corticosteroid use,  n (%) | | 25,035 (33.9) | | 19,984 (33.8) | | 5,051 (34.2) | | 0.712 | 0.399 |  | |
| Comorbidities, n (%) | |  | |  | |  | |  |  |  | |
| Diabetes | | 4,653 (6.3) | | 3,737 (6.3) | | 916 (6.2) | | 0.286 | 0.593 |  | |
| Heart disease | | 8,207 (11.1) | | 6,604 (11.2) | | 1,603 (10.9) | | 1.232 | 0.267 |  | |
| Liver disease | | 4,825 (6.5) | | 3,861 (6.5) | | 964 (6.5) | | 0.000 | 0.985 |  | |
| Renal disease | | 9,104 (12.3) | | 7,275 (12.3) | | 1,829 (12.4) | | 0.046 | 0.830 |  | |
| Hematologic disease | | 4,733 (6.4) | | 3,778 (6.4) | | 955 (6.5) | | 0.088 | 0.767 |  | |
| Malignancy | | 37,214 (50.4) | | 29,748 (50.3) | | 7,466 (50.5) | | 0.173 | 0.678 |  | |
| Organ transplantation | | 3,600 (4.9) | | 2,876 (4.9) | | 724 (4.9) | | 0.022 | 0.881 |  | |
| Asthma | | 926 (1.3) | | 752 (1.3) | | 174 (1.2) | | 0.783 | 0.376 |  | |
| COPD | | 1,095 (1.5) | | 900 (1.5) | | 195 (1.3) | | 3.200 | 0.074 |  | |
| Vital signs, mean (SD) | |  | |  | |  | |  |  |  | |
| Largest variation for BT, ºC | | 0.8 (0.4) | | 0.8 (0.4) | | 0.8 (0.4) | | -0.629 | 0.528 |  | |
| Largest variation for  HR, beats/m | | 16.4 (10.1) | | 16.4 (10.1) | | 16.4 (10.1) | | -0.167 | 0.868 |  | |
| Largest variation for  RR, beats/m | | 2.4 (4.3) | | 2.4 (4.3) | | 2.3 (4.2) | | 0.455 | 0.641 |  | |
| Largest variation for  SBP (mmHg) | | 23.1 (12.6) | | 23.1 (12.6) | | 23.1 (12.5) | | 0.424 | 0.670 |  | |
| Largest variation for  DBP (mmHg) | | 16.5 (8.4) | | 16.5 (8.4) | | 16.4 (8.3) | | 1.465 | 0.139 |  | |
| Laboratory test results, mean (SD) | | | |  | |  | |  |  |  | |
| RBC count (10^3^/Μl) | | 3.7 (0.7) | | 3.7 (0.7) | | 3.7 (0.7) | | -0.215 | 0.83 |  | |
| Haemoglobin (g/Dl) | | 11.3 (2.0) | | 11.3 (2.0) | | 11.3 (2.0) | | -0.353 | 0.725 |  | |
| WBC count (10^3^/Μl) | | 7.6 (4.5) | | 7.5 (4.5) | | 7.6 (4.5) | | -1.661 | 0.097 |  | |
| Platelet count (10^3^/Μl) | | 221.0 (108.6) | | 220.7 (108.6) | | 222.0 (108.3) | | -1.314 | 0.188 |  | |
| Haematocrits (%) | | 33.7 (5.8) | | 33.7 (5.8) | | 33.7 (5.8) | | -0.392 | 0.697 |  | |
| RDW (%) | | 14.6 (2.2) | | 14.6 (2.2) | | 14.6 (2.2) | | 0.334 | 0.741 |  | |
| DNI (%) | | 1.4 (3.2) | | 1.5 (3.3) | | 1.4 (3.0) | | 1.285 | 0.177 |  | |
| Neutrophil count (10^3^/Μl) | | 5.5 (4.1) | | 5.5 (4.1) | | 5.6 (4.1) | | -1.787 | 0.075 |  | |
| Lymphocyte count (10^3^/Μl) | | 1.3 (0.7) | | 1.3 (0.7) | | 1.3 (0.7) | | -0.449 | 0.649 |  | |
| NLR | | 7.1 (48.2) | | 6.9 (35.8) | | 7.7 (80.7) | | -1.864 | 0.224 |  | |
| PNR | | 58.8 (145.5) | | 59.0 (150.6) | | 58.1 (123.5) | | 0.688 | 0.439 |  | |
| PLR | | 237.8 (356.5) | | 237.4 (357.8) | | 239.4 (351.2) | | -0.600 | 0.544 |  | |
| Sodium (mmol/L) | | 138.9 (3.9) | | 138.9 (3.9) | | 138.9 (3.9) | | 0.323 | 0.748 |  | |
| Potassium (mmol/L) | | 4.0 (0.5) | | 4.0 (0.5) | | 4.0 (0.5) | | -0.496 | 0.617 |  | |
| Cl (mmol/L) | | 102.7 (4.4) | | 102.8 (4.4) | | 102.7 (4.4) | | 1.211 | 0.227 |  | |
| CO2 (mmol/L) | | 23.8 (3.3) | | 23.8 (3.3) | | 23.8 (3.3) | | -0.953 | 0.341 |  | |
| Calcium (mmol/L) | | 8.5 (0.7) | | 8.5 (0.7) | | 8.5 (0.7) | | -0.934 | 0.347 |  | |
| Albumin (mmol/L) | | 3.4 (0.6) | | 3.4 (0.6) | | 3.4 (0.6) | | -0.619 | 0.538 |  | |
| Total bilirubin (mmol/L) | | 1.0 (1.9) | | 1.0 (1.9) | | 1.0 (1.9) | | -0.108 | 0.914 |  | |
| BUN (mg/Dl) | | 17.3 (13.5) | | 17.3 (13.5) | | 17.2 (13.4) | | 1.285 | 0.195 |  | |
| Creatinine (mg/Dl) | | 1.1 (1.3) | | 1.1 (1.3) | | 1.1 (1.3) | | 0.113 | 0.911 |  | |
| ALT (mmol/L) | | 36.0 (98.9) | | 36.1 (91.1) | | 35.4 (121.4) | | 0.857 | 0.469 |  | |
| AST (mmol/L) | | 41.4 (163.4) | | 41.4 (125.8) | | 41.5 (262.5) | | -0.090 | 0.952 |  | |
| Radiology test result, n (%) | | | |  | |  | |  |  |  | |
| Chest X-ray, Normal | | 25,121 (34) | | 29,196 (34.2) | | 4,925 (33.3) | |  |  |  | |
| Abnormal | | 42,027 (56.9) | | 33,542 (56.8) | | 8,484 (57.4) | |  |  |  | |
| None | | 6,711 (9.1) | | 5,348 (9.1) | | 1,363 (9.2) | | 3.770 | 0.152 |  | |
| Room status, n (%) | |  | |  | |  | |  | |  |  |
| Same room | | 1,542 (2.1) | | 1,250 (2.1) | | 292 (2.0) | | 1.047 | 0.306 |  | |
| Same unit | | 9,146 (12.4) | | 7,339 (12.4) | | 1,807 (12.2) | | 0.368 | 0.544 |  | |
| Multi-occupancy room | | 64,858 (87.8) | | 51,852 (87.8) | | 13,006 (88.0) | | 0.899 | 0.343 |  | |
| Double room | | 35,534 (48.1) | | 28,450 (48.1) | | 7,084 (48.0) | | 0.170 | 0.680 |  | |

*BMI* Body mass index*, COPD* Chronic obstructive pulmonary disease*, BT* Body temperature*, HR* Heart rate,

*RR* Respiration rate, *SBP* Systolic blood pressure, *DBP* Diastolic blood pressure, *RBC* Red blood cell,

*WBC* White blood cell, *RDW* Red blood cell distribution width, *DNI* Delta neutrophil index,

*NLR* Neutrophil-to-lymphocyte ratio, *PNR* Platelet-to-neutrophil ratio, *PLR* Platelet-to-lymphocyte ratio,

*Cl* Chloride, *BUN* Blood urea nitrogen, *ALT* Alanine transaminase, *AST* Aspartate transaminase

Feature A.1. Feature importance of LR


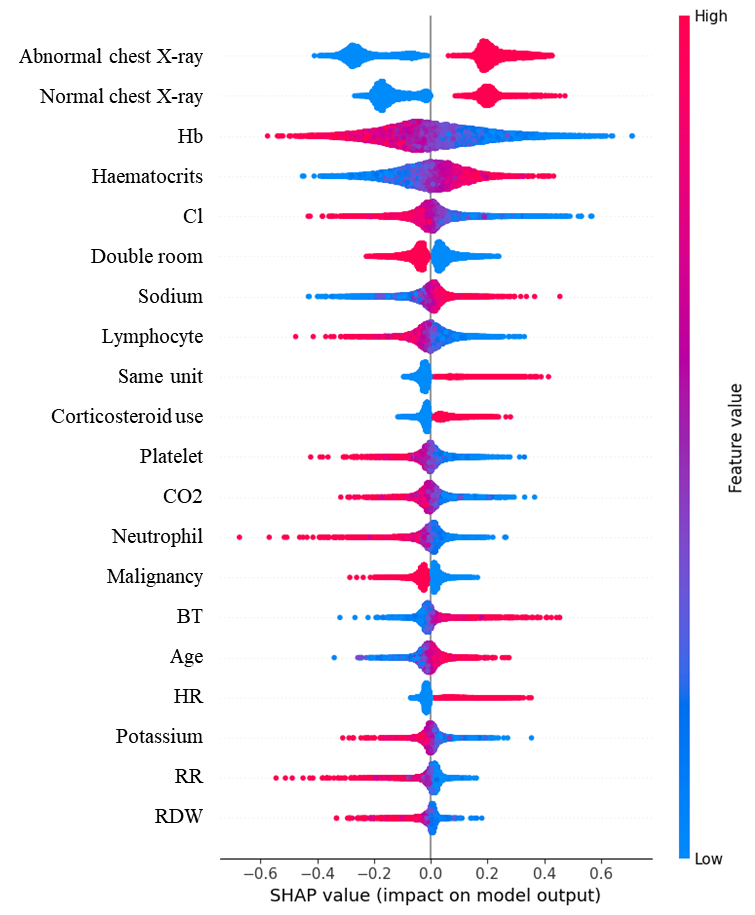


*Hb* Haemoglobin*, Cl* Chloride, *BT* Body temperature, *HR* Heart rate, *RR* Respiration rate,

*RDW* Red blood cell distribution width

Feature A.2. Feature importance of XGB


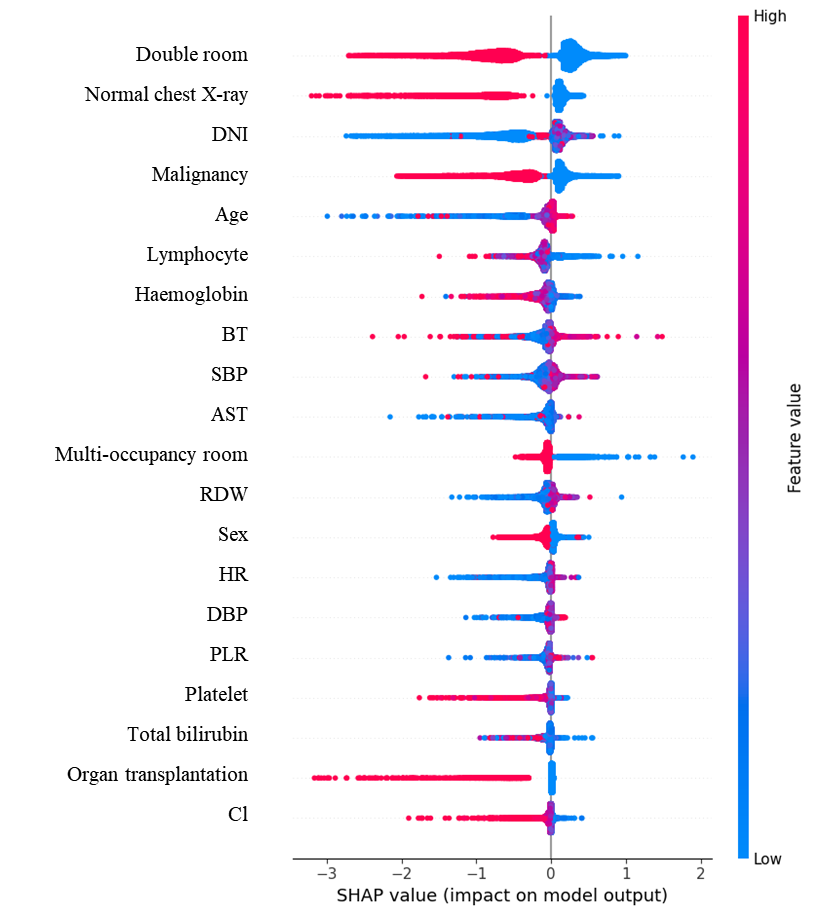


*DNI* Delta neutrophil index, *BT* Body temperature, *SBP* Systolic blood pressure, *AST* Aspartate transaminase, *RDW* Red blood cell distribution width, *HR* Heart rate, *DBP* Diastolic blood pressure,

*PLR* Platelet-to-lymphocyte ratio, *Cl* Chloride

Feature A.3. Feature importance of ANN


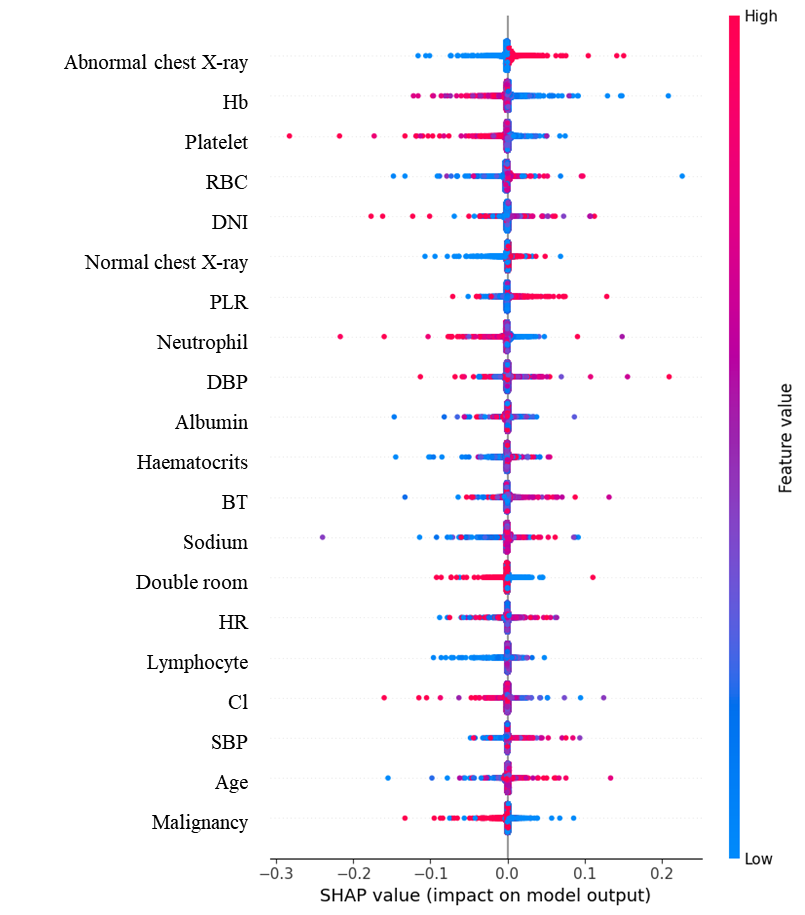


*Hb* Haemoglobin*, RBC* Red blood cell, *DNI* Delta neutrophil index, *PLR* Platelet-to-lymphocyte ratio,

*DBP* Diastolic blood pressure, *BT* Body temperature, *HR* Heart rate, *Cl* Chloride, *SBP* Systolic blood pressure
